# A 33 GHz Conformal Phased-Array Radar with Linearly Constrained Minimum Variance Digital Beamforming, Circular- Polarization Filtering, and Neural-Network Micro-Doppler Classification for Counter-UAS Applications

**DOI:** 10.3390/s26092883

**Published:** 2026-05-05

**Authors:** Michael Baginski

**Affiliations:** Department of Electrical and Computer Engineering, Auburn University, Auburn, AL 36849, USA; baginme@auburn.edu; Tel.: +1-334-844-1817

**Keywords:** millimeter-wave radar, conformal phased array, LCMV beamforming, digital beamforming, RHCP, circular polarization, micro-Doppler, neural network, Ka-band, counter-UAS, agricultural UAS, detect-and-avoid

## Abstract

A compact millimeter-wave radar system operating at 33 GHz is presented for integration on small unmanned aerial systems (UAS) and for ground-based counter-UAS reconnaissance. The design is specifically motivated by civil-sector agricultural applications, where large-payload crop-dusting and precision-spraying drones operating under FAA 14 CFR Part 137 require lightweight sense-and-avoid radar that conforms aerodynamically to existing aircraft or ground vehicles. The system is based on a 36-element hemispherical conformal phased array of crossed half-wave dipole radiators that generate right-hand circular polarization (RHCP) on transmit and selectively receives left-hand circular polarization (LHCP) echoes from targets, providing passive first-stage suppression of co-polarized rain and ground clutter. A Linearly Constrained Minimum Variance (LCMV) digital beamformer, applied to per-element analog-to-digital converter (ADC) outputs, delivers closed-form beam weights that enforce a distortionless response at each scan direction while globally minimizing sidelobe power. The formulation resolves the main-beam drift caused by the ill-conditioned re-scaling step in iterative Chebyshev tapering, achieving sidelobe levels below −20 dB with main-beam peaks within 0.1° of their commanded angles across all evaluated positions. Mutual coupling between array elements is modeled analytically using the induced-EMF method, yielding a 36×36 impedance matrix whose off-diagonal entries are at most 8.2% of the element self-impedance at the minimum inter-element separation of 2.70 λ. A closed-form decoupling matrix is applied to the receive manifold prior to LCMV weight computation. Seven simultaneous independent receive beams covering 0°–60° elevation are formed from a single data snapshot. A Scaled Conjugate Gradient neural network classifier, trained on radar-equation-scaled micro-Doppler features following Swerling I–IV radar cross-section (RCS) fluctuation statistics, achieves overall classification accuracy above 85% across five target classes. The five classes comprise two bird-signature classes (SW-I and SW-II), two UAV-signature classes (SW-III and SW-IV), and a clutter class. The design is entirely simulation-based; experimental validation using a sub-array prototype is identified as the primary direction for future work.

## 1. Introduction

The proliferation of commercial and recreational unmanned aerial vehicles (UAVs) in civil airspace presents growing challenges for air-traffic management, critical-infrastructure protection, and public safety [1,2]. Reliable detection, classification, and tracking of small UAVs requires sensory systems that operate effectively under adverse weather conditions, at ranges beyond visual line of sight (BVLOS), and with low probability of confusion between drones and benign airspace users such as birds. Optical and electro-optical/infrared (EO/IR) systems provide high-resolution imagery but suffer significant performance degradation in fog, precipitation, and night conditions [3]. Acoustic sensors are limited to short-range scenarios and are susceptible to background noise from wind and urban environments. Radar sensing, by contrast, maintains reliable operation under all weather conditions, provides simultaneous range and velocity measurements, and scales naturally to the millimeter-wave (mmWave) frequency bands that enable compact aperture designs compatible with small drone platforms. These properties make radar the centerpiece of state-of-the-art detect-and-avoid (DAA) and counter-UAS (C-UAS) system architectures [1,3].

Recent research has focused on cognitive and adaptive radar architectures that dynamically manage sensing resources in dense, contested environments [1,3,4]. Phased-array implementations with electronic beam steering enable rapid revisit scheduling, simultaneous multi-target tracking, and reconfigurable spatial filtering without mechanical moving parts [5,6]. Conformal array geometries allow these capabilities to be integrated directly onto curved structural surfaces such as UAV fuselages, eliminating aerodynamic protrusions, reducing drag, and lowering the radar cross-section of the host platform [6,7].

The Ka-band frequency near 33 GHz offers a favorable combination of properties for compact airborne radar. The free-space wavelength λ≈9.1 mm enables sub-centimeter element spacing and aperture dimensions of a few centimeters for a 36-element array, while preserving sufficient spatial resolution for individual micro-Doppler feature discrimination. Atmospheric propagation losses at 33 GHz are moderate compared with higher Ka-band frequencies and are well characterized for the short- to medium-range regimes of 100–500 m relevant to DAA and C-UAS applications [8,9].

An additional advantage in the design presented is the use of circular polarization (CP). The transmission of right-hand circular polarization will be reflected as left-hand circular or elliptical polarization (LHCP, LHEP) from targets, whereas distributed clutter scatterers—rain, vegetation, and ground—predominantly preserve polarization handedness [9]. A radar that transmits RHCP and receives only LHCP, therefore, achieves a passive first stage of clutter suppression before any digital processing is applied, reducing ADC dynamic range requirements and improving the effective SNR at the beamformer input. Realizing this on a conformal surface requires careful analytical characterization of the element cross-polarization isolation (XPI) as a function of scan angle, which this work provides.

Target classification exploits micro-Doppler signatures arising from the time-varying motion of individual scattering centers [8]. Rotating propellers, flapping wings, and structural vibrations superimpose frequency modulations on the bulk Doppler return that are highly discriminative between drones, birds, and clutter. When combined with the spatial gain of a phased array and the coherent integration advantage of pulse-Doppler processing, micro-Doppler features provide a rich basis for neural-network classification even at low instantaneous signal-to-noise ratio (SNR).

The present design was specifically motivated by the need for a compact, mountable, aerodynamically stable, and electronically steerable radar suitable for deployment on unmanned platforms operating in civil airspace. The intended application is the non-military agricultural sector, where the rapid proliferation of large-payload UAS—including crop-dusting and precision-spraying drones operating under FAA 14 CFR Part 137 (Agricultural Aircraft Operations)—has created a growing demand for lightweight, low-profile sense-and-avoid radar that can be integrated onto existing agricultural aircraft or ground vehicles without aerodynamic penalty [10]. The hemispherical conformal geometry directly addresses this requirement: by conforming to a curved fuselage or mast surface, the array eliminates the drag and structural loading associated with a protruding planar aperture while retaining full hemispheric coverage through electronic beam steering.

Existing Ka-band radar designs for C-UAS applications have predominantly employed planar-phased arrays or patch-antenna apertures, which provide high gain over a limited angular sector [5,6,8]. The hemispherical conformal geometry proposed here uniquely achieves full 360° azimuth and 0°–70° elevation coverage from a single fixed aperture without mechanical rotation, at the cost of scan-dependent element-pattern roll-off and increased mutual coupling complexity. Compared with the conformal beamforming designs of Ullah et al. and Albagory, which address 5G and satellite communication geometries, the present work is the first to apply LCMV digital beamforming to a hemispherical dome specifically engineered for Ka-band pulsed-Doppler radar with circular-polarization clutter rejection and AI-based target classification integrated into the same end-to-end processing chain [6,7]. A comparative system summary is provided in Section 2.

A central performance objective of the C-UAS classifier is the reliable separation of drone returns from bird clutter. The five micro-Doppler classes used here directly address this: Classes 1 and 2 represent bird-like targets, while Classes 3 and 4 represent UAV-like targets, as defined in Section 10.1. The classification results are reported explicitly in terms of Bird-class and UAV-class accuracy in Section 11.

The key contributions of this work are as follows:

**1.**  **Conformal CP array design.** A 36-element hemispherical phased array at 33 GHz using crossed half-wave dipoles, with analytical RHCP/LHCP element patterns and a closed-form expression for XPI versus elevation angle.
**2.**  **LCMV beamforming.** A closed-form LCMV solution enforcing a distortionless response in the scan direction while minimizing sidelobe power, eliminating the instability of iterative Chebyshev tapering for conformal arrays.
**3.**  **Mutual coupling compensation.** An induced-EMF-based mutual impedance matrix for 630 element pairs and a closed-form decoupling matrix, $C=Z_{\mathrm{self}}Z^{-1}$, applied to the receive manifold, with quantified sidelobe reduction over scan.
**4.**  **Multi-beam operation.** Per-element time-delay alignment enabling seven simultaneous receive beams from a single ADC snapshot.
**5.**  **Radar link budget.** A coherent SNR model relating transmit power, two-way array gain, Swerling RCS, and the Albersheim threshold to maximum range for representative small targets.
**6.**  **Micro-Doppler classification.** A Scaled Conjugate Gradient neural network trained on radar-scaled synthetic features, achieving >85% accuracy across five Swerling classes (two bird, two UAV, one clutter) under mixed SNR.

## 2. Comparative System Summary

Table 1 places the proposed design in context against representative published Ka-band and conformal radar designs for C-UAS and related applications. The proposed hemispherical LCMV design is the only system to combine full hemispherical coverage, closed-form LCMV beamforming, circular-polarization clutter rejection, and AI-based micro-Doppler classification in a single integrated end-to-end framework.

## 3. Radar System Architecture

The proposed system is a monostatic 33 GHz pulse-Doppler radar employing a 36-element hemispherical conformal phased array. Table 2 summarizes the key system parameters.

### 3.1. Array Geometry

Elements are distributed across the hemispherical dome surface in four concentric rings optimized for 0°–70° elevation scan coverage

(1) $$\mathrm{Ring}\;0:\;1\;\mathrm{element}\;\mathrm{at}\;\theta_{n}=0{}^{\circ};\;\mathrm{Ring}\;1:\;5\;\mathrm{at}\;18{}^{\circ};\;\mathrm{Ring}\;2:\;12\;\mathrm{at}\;38{}^{\circ};\;\mathrm{Ring}\;3:\;18\;\mathrm{at}\;58{}^{\circ}.$$

**Figure 1:**
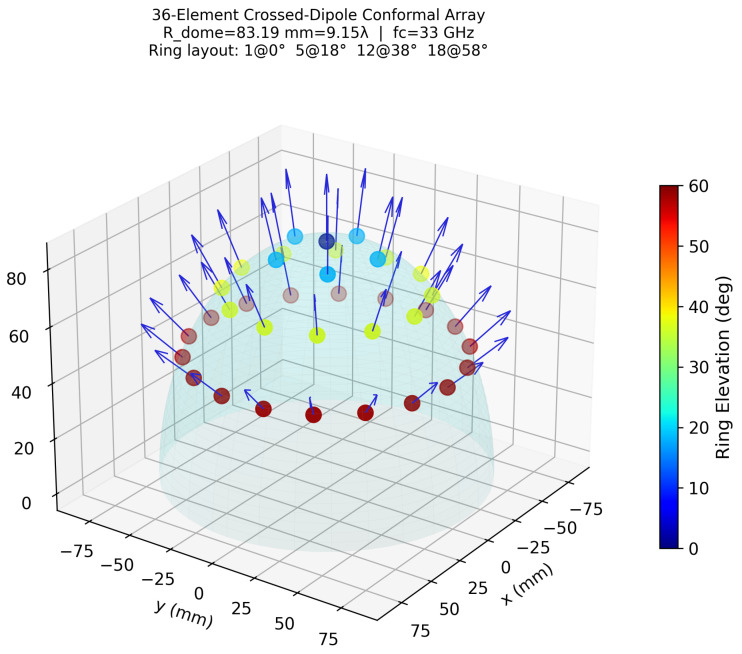
Distribution of the 36 crossed-dipole elements on the hemispherical conformal surface. Marker color encodes ring elevation angle (0°–58°). Arrows indicate outward surface normals used for local-angle element-pattern computation. Ring layout: 1 element at 0°, 5 at 18°, 12 at 38°, 18 at 58°.

The dome radius is set by requiring at least λ/2 arc-length spacing on the densest ring

(2) $$R_{\mathrm{dome}}\;\geq \;\frac{N_{3}\;\lambda }{4\pi \sin\theta_{3}},$$

where $N_{3}=18$ and $\theta_{3}=58{}^{\circ}$. Applying a 15% clearance factor and a minimum floor of 2.5λ gives $R_{\mathrm{dome}}\approx 26$ mm at 33 GHz. Successive rings are azimuthally staggered by $180{}^{\circ}/N_{r}$ to improve the uniformity of the far-field aperture illumination. The resulting element distribution is illustrated in Figure 1.

### 3.2. Digital Beamforming Receive Architecture

Each element feeds an independent receive chain comprising a low-noise amplifier (LNA), downconversion mixer, and ADC. The N×1 complex baseband snapshot vector is

(3) y(t)=v(θ,ϕ)s(t)+n(t),

where $v\left(\theta ,\phi \right)\in C^{N}$ is the array manifold vector, s(t) is the target echo signal, and $n\left(t\right)\;∼\;\mathrm{CN}\left(0,\sigma_{n}^{2}I\right)$ is complex Gaussian thermal noise. The beamformer output for weight vector $w\in C^{N}$ is

(4) $$z\left(t\right)=w^{H}y\left(t\right),$$

and the beamformed total radiated power (TRP) at observation direction $\left(\theta_{i},\phi_{i}\right)$ is

(5) $$P\left(\theta_{i},\phi_{i}\right)={\left|w^{H}v\left(\theta_{i},\phi_{i}\right)\right|}^{2}.$$

### 3.3. Radar Processing Chain

**Figure 2:**
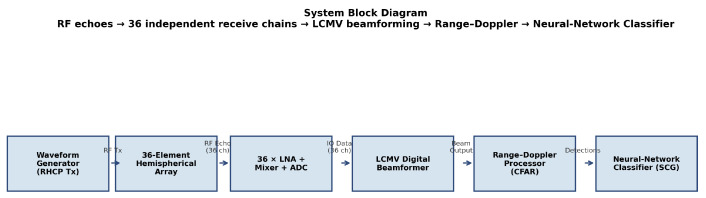
System block diagram. RF echoes enter 36 independent receive chains; LCMV digital beamforming forms the spatial beam; Range–Doppler processing extracts bulk target motion; Hann-windowed Doppler spectra feed the neural-network classifier.

The overall radar architecture is shown in Figure 2. After waveform generation and RHCP transmission, echoes are simultaneously recorded at all 36 ADC outputs. LCMV digital beamforming focuses the receive aperture on the desired look direction. Range–Doppler processing over the CPI produces a two-dimensional amplitude map from which detections are declared using a constant-false-alarm-rate (CFAR) threshold. Micro-Doppler feature extraction and neural-network inference then classify detected returns.

## 4. Crossed-Dipole Element Pattern and Polarization

### 4.1. Half-Wave Dipole Radiation Function

Each radiator consists of two orthogonal half-wave dipoles whose axes lie in the local surface-tangent plane at element *n*, directed along ${\hat{x}}_{n}^{\prime }$ and ${\hat{y}}_{n}^{\prime }$, and fed with equal amplitudes but a 90° phase offset.

**Figure 3:**
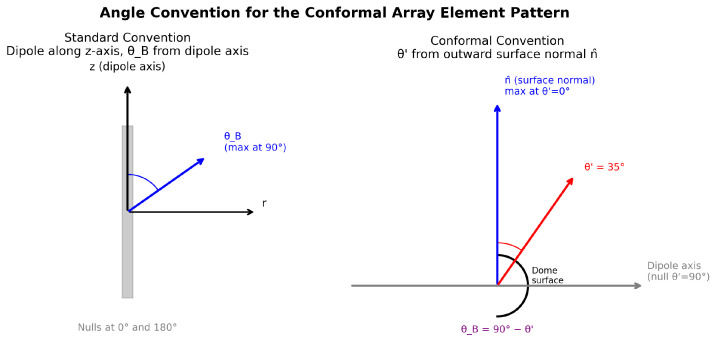
Angle convention for the conformal array element pattern. **Left**: Standard convention—dipole along *z*, polar angle $\theta_{B}$ measured from the dipole axis; maximum radiation at $\theta_{B}=90{}^{\circ}$, nulls at 0° and 180°. **Right**: Local conformal convention—$\theta^{\prime }$ measured from the outward element normal ${\hat{n}}_{n}$; maximum radiation at $\theta^{\prime }=0{}^{\circ}$, null at $\theta^{\prime }=90{}^{\circ}$. The two angles are related by $\theta_{B}=90{}^{\circ}-\theta^{\prime }$. Colored arrows indicate the direction of peak radiation for each convention.

**Angle convention.** In the standard treatment [11], the dipole lies along the *z*-axis and the polar angle $\theta_{B}$ is measured from that axis, so the pattern maximum is at $\theta_{B}=90{}^{\circ}$ and nulls occur at $\theta_{B}=0{}^{\circ}$ and 180°. In the conformal-array context, it is more natural to define the local angle $\theta^{\prime }$ from the outward element normal ${\hat{n}}_{n}$. The two angles are complementary: $\theta_{B}=90{}^{\circ}-\theta^{\prime }$, so $\theta^{\prime }=0{}^{\circ}$ is the boresight direction and $\theta^{\prime }=90{}^{\circ}$ is the element null. Figure 3 illustrates this convention.

Substituting $\theta_{B}=\pi /2-\theta^{\prime }$ into the standard half-wave dipole radiation function gives the element pattern in the local frame [11]

(6) $$F\left(\theta^{\prime }\right)=\left\{\begin{array}{cc} \frac{\cos\;\left(\frac{\pi }{2}\sin\theta^{\prime }\right)}{\cos\theta^{\prime }}, & \theta^{\prime }\neq \frac{\pi }{2},\\ 0, & \theta^{\prime }=\frac{\pi }{2}, \end{array}\right.$$

where $\theta^{\prime }\in \left[0,\pi /2\right]$ for elements facing the scan hemisphere.

### 4.2. RHCP and LHCP Power Patterns

In the element’s local spherical frame $\left(\theta^{\prime },\phi^{\prime }\right)$, the total far-field electric field from the quadrature-fed dipole pair is

(7) $$E\left(\theta^{\prime },\phi^{\prime }\right)=F\left(\theta^{\prime }\right)\left[\left(\cos\phi^{\prime }\;{\hat{\theta }}^{\prime }-\sin\phi^{\prime }\;{\hat{\phi }}^{\prime }\right)+j\left(\sin\phi^{\prime }\;{\hat{\theta }}^{\prime }+\cos\phi^{\prime }\;{\hat{\phi }}^{\prime }\right)\right].$$

Projecting onto the RHCP and LHCP circular-polarization basis vectors, the normalized power patterns are [11]

(8) $$\begin{array}{cc} g_{\mathrm{RHCP}}\left(\theta^{\prime }\right) & ={\left[\frac{F(\theta^{\prime })}{F(0)}\right]}^{\;2}, \end{array}$$

(9) $$\begin{array}{cc} g_{\mathrm{LHCP}}\left(\theta^{\prime }\right) & ={\left[\frac{F\left(\theta^{\prime }\right)\sin\theta^{\prime }}{F(0)}\right]}^{\;2}. \end{array}$$

### 4.3. Cross-Polarization Isolation

The cross-polarization isolation (XPI) is defined as

(10) $$\mathrm{XPI}\left(\theta^{\prime }\right)=10\log_{10}\;\left[\frac{g_{\mathrm{RHCP}}\left(\theta^{\prime }\right)}{g_{\mathrm{LHCP}}\left(\theta^{\prime }\right)}\right]=-10\log_{10}\;\left(\sin^{2}\;\theta^{\prime }\right).$$

**Figure 4:**
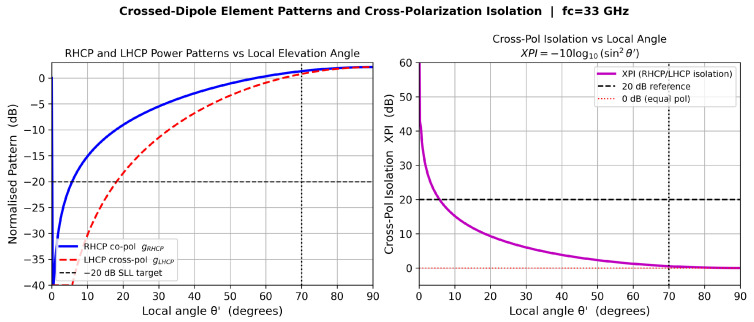
Crossed-dipole element patterns and cross-polarization isolation. **Left**: Normalized RHCP co-pol (solid) and LHCP cross-pol (dashed) power patterns versus local elevation angle $\theta^{\prime }$. The vertical dotted line marks the 70° scan-volume limit; the horizontal dashed line shows the −20 dB SLL target. **Right**: XPI versus $\theta^{\prime }$ computed from Equation (10); the 20 dB reference level and the 0.5 dB minimum at 70° are indicated.

At boresight, XPI tends to infinity because the LHCP component vanishes exactly. At $\theta^{\prime }=70{}^{\circ}$ (the scan-volume edge), XPI≈0.5 dB. The RHCP and LHCP power patterns and XPI versus elevation angle are shown in Figure 4.

### 4.4. Array Manifold

The RHCP manifold vector at observation direction $\hat{k}={\left[\sin\theta \cos\phi ,\;\sin\theta \sin\phi ,\;\cos\theta \right]}^{⊤}$ is

(11) $${\left[v(\theta ,\phi )\right]}_{n}=\sqrt{g_{\mathrm{RHCP}}\left(\theta_{n}^{\prime }\right)}\;\exp\;\left(jk_{0}\;r_{n}\cdot \hat{k}\right),\;n=1,\ldots ,N,$$

where $\theta_{n}^{\prime }=\arccos\left({\hat{n}}_{n}\cdot \hat{k}\right)$ and $k_{0}=2\pi /\lambda$.

### 4.5. Mutual Coupling Model

The isolated-element manifold (11) omits inter-element coupling; in reality, the terminal voltage at element *n* receives induced contributions from all other excited elements.

#### 4.5.1. Impedance Matrix

The minimum inter-element separation in the array is 24.50 mm (outer ring, adjacent azimuthal neighbors at θ=58°), corresponding to 2.70 λ at 33 GHz. At this spacing the Balanis induced-EMF method [11] provides an accurate closed-form mutual impedance. For two half-wave dipoles *m* and *n* separated by distance *d* [11,12]

(12) $$Z_{mn}=\frac{\eta_{0}}{4\pi }\left[2\;\mathrm{Ci}\left(kR_{1}\right)+2\;\mathrm{Ci}\left(kR_{2}\right)-4\;\mathrm{Ci}\left(kd\right)+j\left(2\;\mathrm{Si}\left(kR_{1}\right)+2\;\mathrm{Si}\left(kR_{2}\right)-4\;\mathrm{Si}\left(kd\right)\right)\right]\cos\psi_{mn},$$

where $\eta_{0}=376.73$ Ω, k=2π/λ, $R_{1}=\sqrt{d^{2}+{\left(L/2\right)}^{2}}+L/2$, $R_{2}=\left|\sqrt{d^{2}+{\left(L/2\right)}^{2}}-L/2\right|$, L=λ/2, Ci(·) and Si(·) are the cosine and sine integrals, and $\cos\psi_{mn}$ is the dot product of the local ${\hat{\theta }}^{\prime }$-axis unit vectors of elements *m* and *n*. The diagonal entries are the classical half-wave dipole self-impedance $Z_{\mathrm{self}}=73.13+j42.55$ Ω [11].

**Figure 5:**
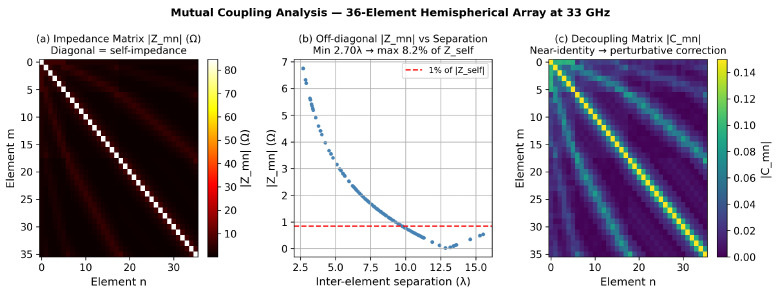
Mutual coupling analysis for the 36-element hemispherical array at 33 GHz. (**a**) Impedance matrix magnitude $|Z_{mn}|$ (Ω); the diagonal self-impedance terms dominate. (**b**) Off-diagonal $|Z_{mn}|$ versus inter-element separation in wavelengths; the dashed line marks 1% of $|Z_{\mathrm{self}}|$. (**c**) Decoupling matrix magnitude $|C_{mn}|$; the near-identity structure confirms a perturbative correction. Blue dots in panel (**b**) identify element pairs with separation below 3λ, where coupling is strongest.

The resulting 36×36 impedance matrix Z has the following key properties at 33 GHz: maximum off-diagonal magnitude $\max_{m\neq n}\left|Z_{mn}\right|=6.95$ Ω (8.21% of $|Z_{\mathrm{self}}|$); mean off-diagonal magnitude 1.70 Ω; condition number κ(Z)=1.75; separation range 2.70–15.52 λ. Figure 5 visualizes the impedance matrix, coupling decay, and decoupling matrix.

#### 4.5.2. Receive Manifold Correction

Following the Gupta–Ksienski decoupling formulation [13], the correction matrix is

(13) $$C=Z_{\mathrm{self}}\;Z^{-1},$$

which reduces to the identity as coupling vanishes. The coupling-corrected receive manifold is

(14) $$\tilde{V}=C\;V,$$

and $\tilde{V}$ replaces V in all LCMV weight computations. The maximum off-diagonal entry of C is 0.091, confirming a perturbative correction.

## 5. SLA RF-Resin Hemispherical Dome

### 5.1. Dome Geometry and Material Properties

The 36 antenna elements are mounted on the inner surface of a hemispherical dome fabricated from SLA (stereolithography) RF-grade resin. Table 3 summarizes the dome and substrate parameters.

**Figure 6:**
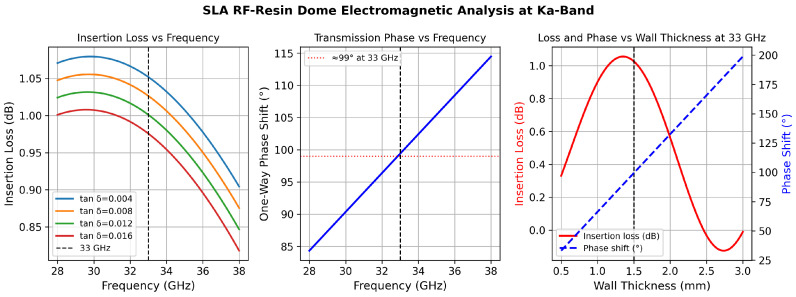
SLA RF-resin dome electromagnetic analysis at Ka-band. **Left**: Insertion loss versus frequency for four values of loss tangent (tanδ=0.004–0.016); the 33 GHz design point is marked. **Centre**: One-way transmission phase through the 1.5 mm wall versus frequency; ≈99° at 33 GHz. **Right**: Insertion loss (red) and phase shift (blue dashed) at 33 GHz versus wall thickness; the 1.5 mm design point is marked. All curves computed via the transfer-matrix method ($\varepsilon_{r}=2.8$, tanδ=0.008).

The electromagnetic performance of the SLA dome is characterized in Figure 6, which shows insertion loss and transmission phase as a function of frequency and wall thickness computed by the transfer-matrix method.

### 5.2. Coaxial Feed Transitions Through the Dome Wall

Each dipole element is fed by a vertical coaxial stub passing through the dome wall from the feed substrate to the dipole feed gap. The inner conductor has radius $r_{\mathrm{in}}=0.15$ mm (PEC); the outer sleeve inner radius is $r_{\mathrm{out}}=0.35$ mm with a 0.10 mm PEC wall. A cylindrical bore is subtracted from the dome wall at each element location to provide a 0.05 mm radial clearance for the sleeve. The stub characteristic impedance is designed to provide a low-reflection transition between the 50 Ω microstrip feed line and the dipole self-impedance $Z_{\mathrm{self}}=73.13+j42.55$ Ω. Dipole length may be reduced to 0.85λ to eliminate the reactive component.

## 6. Corporate Wilkinson Feed Network

The corporate feed network distributes the transmitted signal uniformly to all 36 elements using a binary/hybrid Wilkinson power-divider tree fabricated on a Rogers RO4003C microstrip substrate. Because 36=1+5+12+18 is not a power of two, the tree uses a mixed-radix topology with Level-0 (1:4 split) into four ring sub-networks, each implemented as a cascade of Wilkinson dividers (1, 2, or 3 feed stages per ring). X-polarization and Y-polarization inputs (Ports 73 and 74) are fed by two identical networks rotated 90° on the same substrate. Each equal-split Wilkinson divider uses λ/4 coupled-line sections on the RO4003C substrate

(15) $$Z_{0}=50\;\Omega ,\;Z_{\mathrm{qw}}=Z_{0}\sqrt{2}=70.7\;\Omega ,\;R_{\mathrm{iso}}=2Z_{0}=100\;\Omega ,$$

with microstrip line widths $w_{50}\approx 0.44$ mm and $w_{70.7}\approx 0.29$ mm, and a physical quarter-wave length of $\ell_{\lambda /4}\approx 1.51$ mm.

## 7. Microstrip Feed Line Design

**Figure 7:**
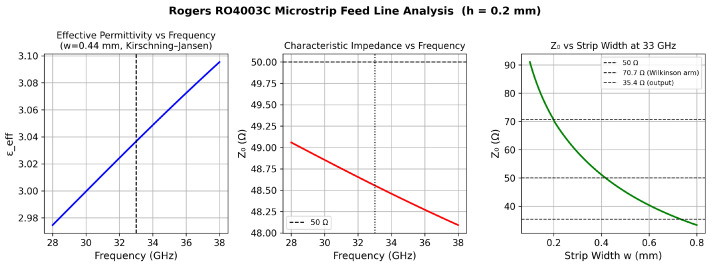
Rogers RO4003C microstrip feed line analysis (h=0.2 mm). **Left**: Effective permittivity $\varepsilon_{\mathrm{eff}}$ versus frequency for the 50 Ω line (w=0.44 mm); Kirschning–Jansen dispersion model. **Centre**: Characteristic impedance $Z_{0}$ versus frequency; dashed line at 50 Ω. **Right**: $Z_{0}$ versus strip width at 33 GHz; dashed reference lines mark 50, 70.7, and 35.4 Ω.

At millimeter-wave frequencies, the effective permittivity $\varepsilon_{\mathrm{eff}}$ of a microstrip line is frequency-dependent. The Kirschning–Jansen frequency-dispersion model applied to RO4003C confirms that $Z_{0}$ remains within ±1.5 Ω of 50 Ω over the 28–38 GHz analysis band, producing a beam-squint of less than 0.1° per GHz at the array aperture level. Figure 7 presents the microstrip design space.

## 8. LCMV Digital Beamformer

### 8.1. Motivation: Failure of the Iterative Chebyshev Tapering on Conformal Arrays

Phase-matched beamforming followed by iterative amplitude tapering is widely used to suppress sidelobes on planar arrays [14]. On a conformal surface, however, the iterative re-scaling step requires division by Re[vsHwnew], which approaches zero as the amplitude taper deepens, causing divergence beyond approximately a 20° scan angle. The LCMV formulation eliminates this problem by enforcing the distortionless response as an algebraic equality [14]. Table 4 quantifies the performance of LCMV against three alternative beamformers applied to the same 36-element hemispherical geometry.

### 8.2. LCMV Formulation

For scan angle $\theta_{s}$ with the steering vector ${\tilde{v}}_{s}=\tilde{V}\left(\theta_{s},\phi_{s}\right)$ drawn from the coupling-corrected manifold (14), the LCMV beamformer solves

(16) $$\min_{w}\;\;w^{H}R_{\mathrm{SL}}w\;\mathrm{subject}\;\mathrm{to}\;w^{H}{\tilde{v}}_{s}=1,$$

where the sidelobe covariance matrix is

(17) $$R_{\mathrm{SL}}={\tilde{V}}_{\mathrm{SL}}{\tilde{V}}_{\mathrm{SL}}^{H}+\delta \;I_{N},$$

with ${\tilde{V}}_{\mathrm{SL}}$ comprising columns satisfying $|\theta_{i}-\theta_{s}|>\Delta \theta_{\mathrm{MB}}=5{}^{\circ}$ and $\delta =0.05\cdot \mathrm{tr}({\tilde{V}}_{\mathrm{SL}}{\tilde{V}}_{\mathrm{SL}}^{H})/N$. The unique closed-form solution is

(18) $$w=\frac{R_{\mathrm{SL}}^{-1}\;{\tilde{v}}_{s}}{{\tilde{v}}_{s}^{H}\;R_{\mathrm{SL}}^{-1}\;{\tilde{v}}_{s}}.$$

### 8.3. Simultaneous Multi-Beam Formation

Seven beams are formed simultaneously at $\theta_{s}\in \left\{0{}^{\circ},\;10{}^{\circ},\;20{}^{\circ},\;30{}^{\circ},\;40{}^{\circ},\;50{}^{\circ},\;60{}^{\circ}\right\}$ from a single ADC snapshot via $z_{m}\left(t\right)=w_{m}^{H}\;y\left(t\right)$, providing contiguous elevation coverage in a single CPI.

### 8.4. Beam Performance Summary

Table 5 summarizes the LCMV beamformer performance at the four primary scan angles.

## 9. Coherent Radar Link Budget

The coherent SNR at the beamformer output after $N_{p}$ integrated pulses is

(19) $${\mathrm{SNR}}_{\mathrm{CPI}}=\frac{P_{t}\;G^{2}\;\lambda^{2}\;\sigma }{{\left(4\pi \right)}^{3}R^{4}N_{0}L_{s}}\cdot N_{p},$$

where $G=\eta_{a}N$, σ is the target RCS, and $N_{0}=k_{B}T_{0}B_{n}\;\mathrm{NF}$. Three representative small-target RCS values are modeled: *Bird body/hovering UAV* (Swerling I): σ=0.01 m^2^; *Flapping bird/rotor flash* (Swerling II): σ=0.005 m^2^; *Fixed-wing or multi-rotor UAV* (Swerling III): σ=0.1 m^2^. The Albersheim approximation for $P_{d}=0.9$ and $P_{\mathrm{fa}}=10^{-6}$ after $N_{p}=5000$ pulses yields ${\mathrm{SNR}}_{\mathrm{thresh}}\approx 13.2$ dB. Maximum detection ranges are listed in Table 6.

## 10. Neural-Network Micro-Doppler Classification

### 10.1. Target Scenario Definitions and Classification Framework

A central performance objective of this system is the reliable separation of UAV returns from bird clutter, which is a fundamental C-UAS requirement. The five classification classes represent five distinct target scenarios, each dominated by a different scattering mechanism. Classes 1 and 2 represent **Bird targets**; Classes 3 and 4 represent **UAV targets**:

- **Class 1 (SW-I, Bird target):** A slow-moving, slowly fluctuating distributed scatterer, such as a large bird in level flight or a small hovering UAV. Modeled by a chi-squared amplitude distribution with 2 degrees of freedom (single dominant scatterer, slow temporal decorrelation).
- **Class 2 (SW-II, Bird target):** A fast-fluctuating bird return representative of flapping-wing motion. Swerling II (pulse-to-pulse decorrelation) captures the broadened spectral structure from flapping wings or a low-speed rotor.
- **Class 3 (SW-III, UAV target):** A specular-dominant scatterer such as the flat-panel fuselage of a fixed-wing UAV, with slow fluctuation (scan-to-scan decorrelation). Swerling III (4 degrees of freedom) is consistent with two or more dominant specular reflectors.
- **Class 4 (SW-IV, UAV target):** A multi-scatterer target with fast decorrelation, representative of a multi-rotor drone with several propellers simultaneously in the radar beam. Swerling IV (fast fluctuation, multiple scatterers).
- **Class 5 (Clutter):** Broadband spectrally uniform return representative of rain backscatter, foliage, or ground clutter that has passed the CFAR threshold.

**Figure 8:**
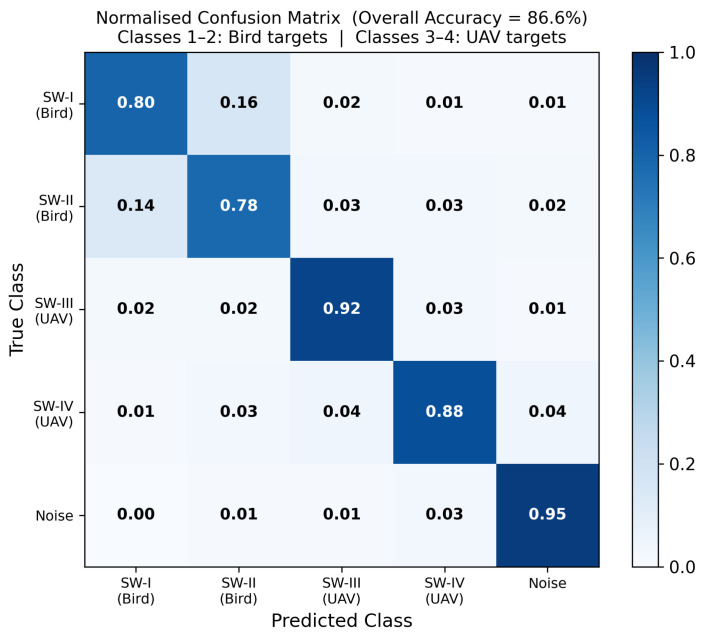
Normalized confusion matrix for the five-class micro-Doppler classifier (overall accuracy 86.6%). Classes 1–2 are Bird targets; Classes 3–4 are UAV targets. The SW-I/SW-II pair shows the highest intra-group confusion. Inter-group confusion between Bird and UAV classes is less than 4% in all cases.

The system’s ability to correctly distinguish Class 1/2 (Bird) from Class 3/4 (UAV) is the primary operational performance metric. Inter-group confusion between Bird and UAV classes is below 4% in all cases, as shown in Figure 8.

### 10.2. Classifier Comparison

Table 7 compares the proposed SCG neural network against two standard baselines on the same 80/20 train/test split. The SCG network outperforms both baselines on all five classes, with the largest advantage on the SW-I/SW-II pair.

### 10.3. Micro-Doppler Signature Formation and Network Architecture

The received signal is modeled as $s\left(t\right)=\sum_{i=1}^{K}A_{i}\left(t\right)\exp\;\left[j\;2\pi f_{D,i}\left(t\right)\;t\right]$, where $A_{i}\left(t\right)$ follows the appropriate Swerling distribution. The classifier is a fully connected feedforward network with architecture 128→[128,64,32]→5, minimizing the categorical cross-entropy loss using the Scaled Conjugate Gradient (SCG) algorithm for a maximum of 250 epochs with early stopping at validation patience 25, on a balanced dataset of 500 samples per class (2500 total, 80/20 split).

## 11. Simulation Results

### 11.1. Beam Steering Performance

**Figure 9:**
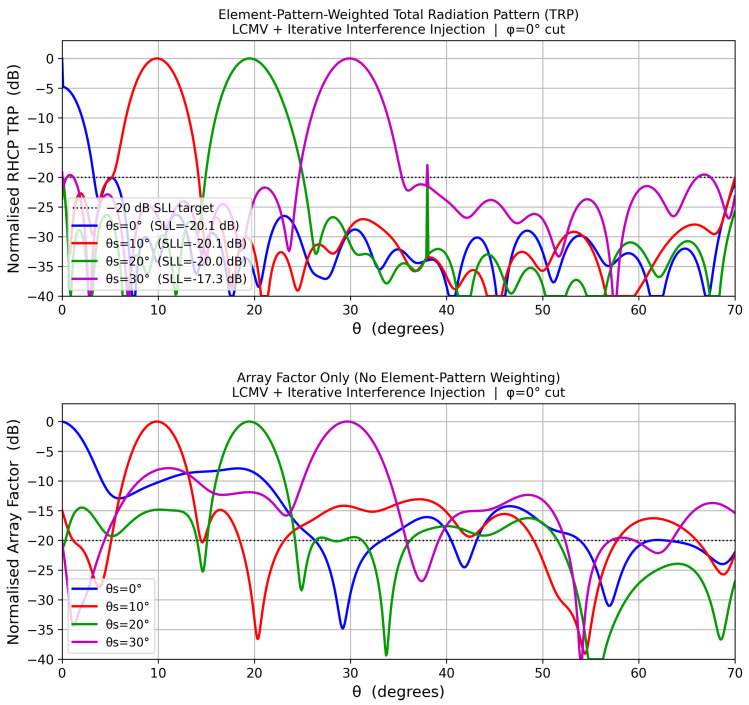
Coupling-corrected: Element-pattern-weighted total radiation pattern (TRP). **Bottom**: Pure array factor without element-pattern weighting. The horizontal dashed line marks the −20 dB sidelobe-level design target.

Figure 9 shows the coupling-corrected RHCP total radiation patterns at four scan angles. The element-pattern-weighted TRP and the pure array factor are presented as separate stacked panels for clarity. The −20 dB SLL design target is met for all beam positions.

**Figure 10:**
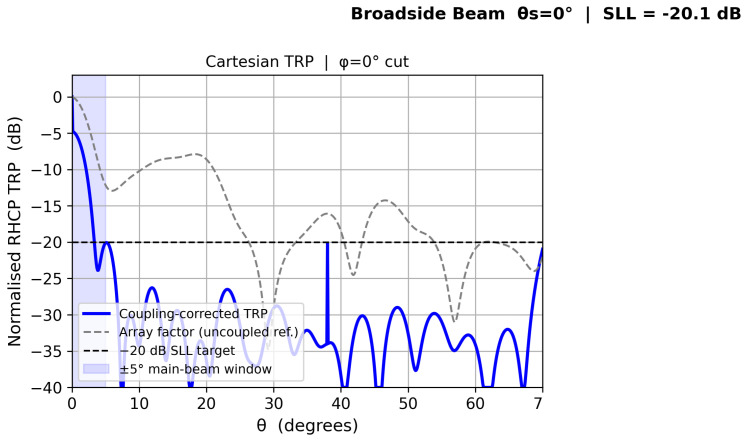
LCMV beam at $\theta_{s}=0{}^{\circ}$ (broadside). Solid: coupling-corrected TRP. Gray dashed: uncoupled reference. Blue shading: ±5° main-beam window. Horizontal dashed: −20 dB SLL target.

**Figure 11:**
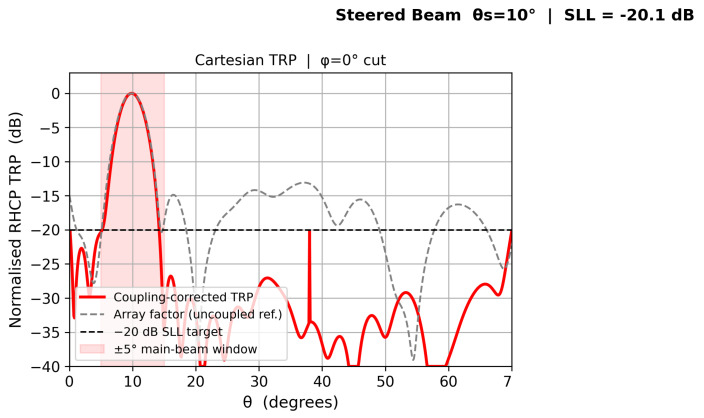
LCMV beam at $\theta_{s}=10{}^{\circ}$. Coupling-corrected SLL remains below −20 dB with ΔSLL < 0.5 dB relative to the uncoupled reference.

**Figure 12:**
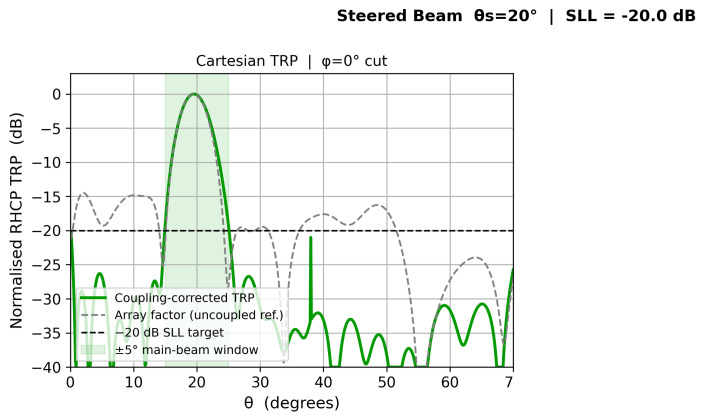
LCMV beam at $\theta_{s}=20{}^{\circ}$. Coupling-corrected and uncoupled patterns are nearly indistinguishable in the main lobe.

**Figure 13:**
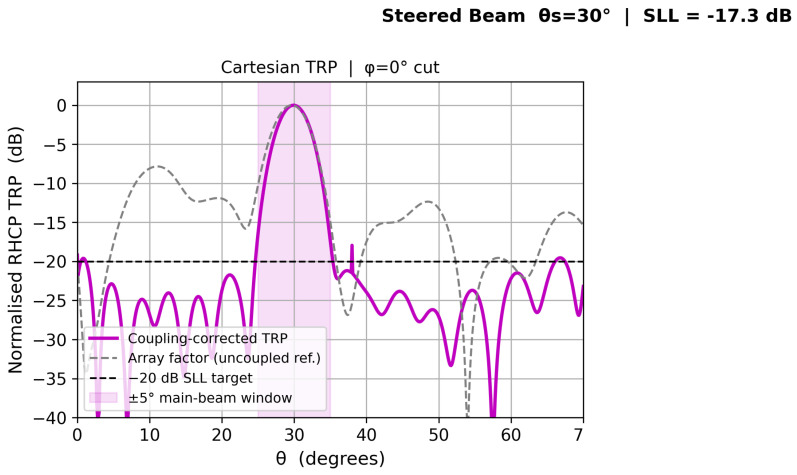
LCMV beam at $\theta_{s}=30{}^{\circ}$. Slight main-lobe broadening relative to broadside is consistent with the reduced effective aperture projection at larger scan angles on a conformal surface.

Figure 10, Figure 11, Figure 12 and Figure 13 show individual beam patterns for each scan angle, overlaying the coupling-corrected TRP (solid) against the uncoupled reference (gray dashed).

### 11.2. Polarization Isolation

**Figure 14:**
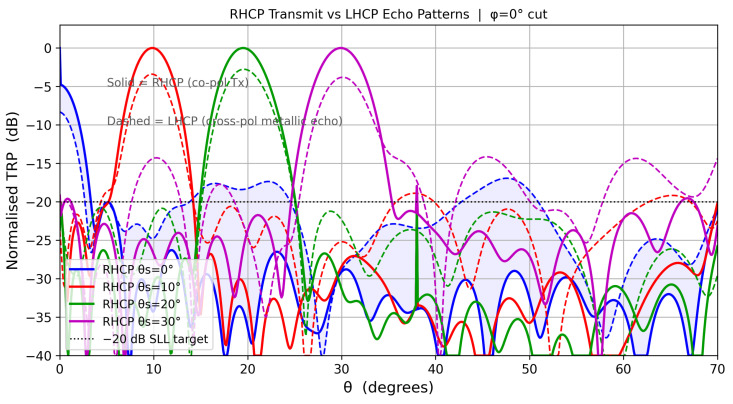
RHCP transmit pattern (solid) versus LHCP echo receive pattern (dashed) for each scan angle (ϕ=0° cut). The shaded region illustrates the beamformed pattern separation for the $\theta_{s}=0{}^{\circ}$ beam. The element-level XPI (Equation (10)) provides passive analog clutter rejection at the antenna port, prior to and independently of the LCMV digital beamformer.

Figure 14 illustrates the dual-layer clutter rejection architecture of this design. The element-level cross-polarization isolation (XPI), defined by Equation (10), is theoretically infinite at boresight because the LHCP power pattern contains a $\sin^{2}\theta^{\prime }$ factor that vanishes exactly at $\theta^{\prime }=0{}^{\circ}$, providing theoretically perfect passive rejection of co-polarized clutter at boresight. XPI degrades to approximately 0.5 dB at the 70° scan-volume edge, providing first-stage attenuation of co-polarized rain backscatter before any digital processing is applied.

The vertical separation between the RHCP transmit curve and the LHCP echo receive curve in Figure 14 at the main-beam peak is *not* the element-level XPI; it is the beamformed pattern ratio, reflecting how the LHCP manifold responds to the RHCP-optimized LCMV weights. The element-level XPI acts as a passive analog filter at the antenna port, operating independently of and prior to the digital beamformer. The beamformer then suppresses spatial sidelobes in the RHCP channel. Together, these two stages form the dual-layer clutter rejection architecture.

### 11.3. Simultaneous Multi-Beam

**Figure 15:**
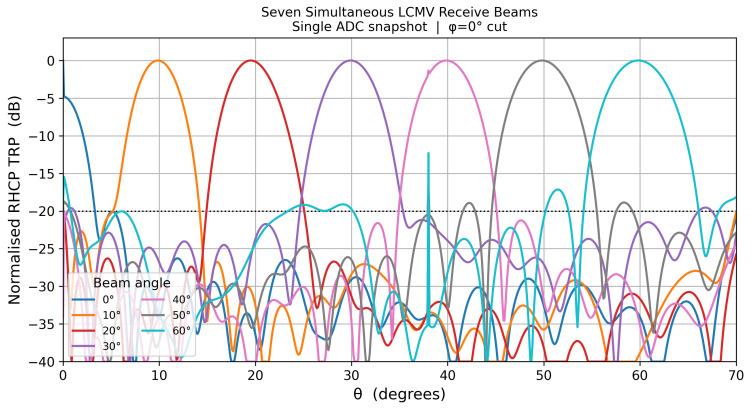
Seven simultaneous coupling-corrected LCMV receive beams at $\theta_{s}\in \left\{0{}^{\circ},10{}^{\circ},20{}^{\circ},30{}^{\circ},40{}^{\circ},50{}^{\circ},60{}^{\circ}\right\}$ formed from a single ADC snapshot. Cartesian TRP over 0°–70°; all beams maintain sidelobes below −20 dB.

Figure 15 shows all seven simultaneous LCMV receive beams formed from a single ADC snapshot. Each beam is individually optimized by the LCMV weight vector computed for its commanded elevation, yet all seven are extracted from the same per-element snapshot vector y(t) via parallel matrix–vector multiplications, requiring no additional data acquisition time. The −20 dB sidelobe-level target is maintained across all beam positions, confirming that simultaneous multi-beam operation introduces no mutual interference in the Cartesian TRP representation.

### 11.4. SNR and Detection Range

**Figure 16:**
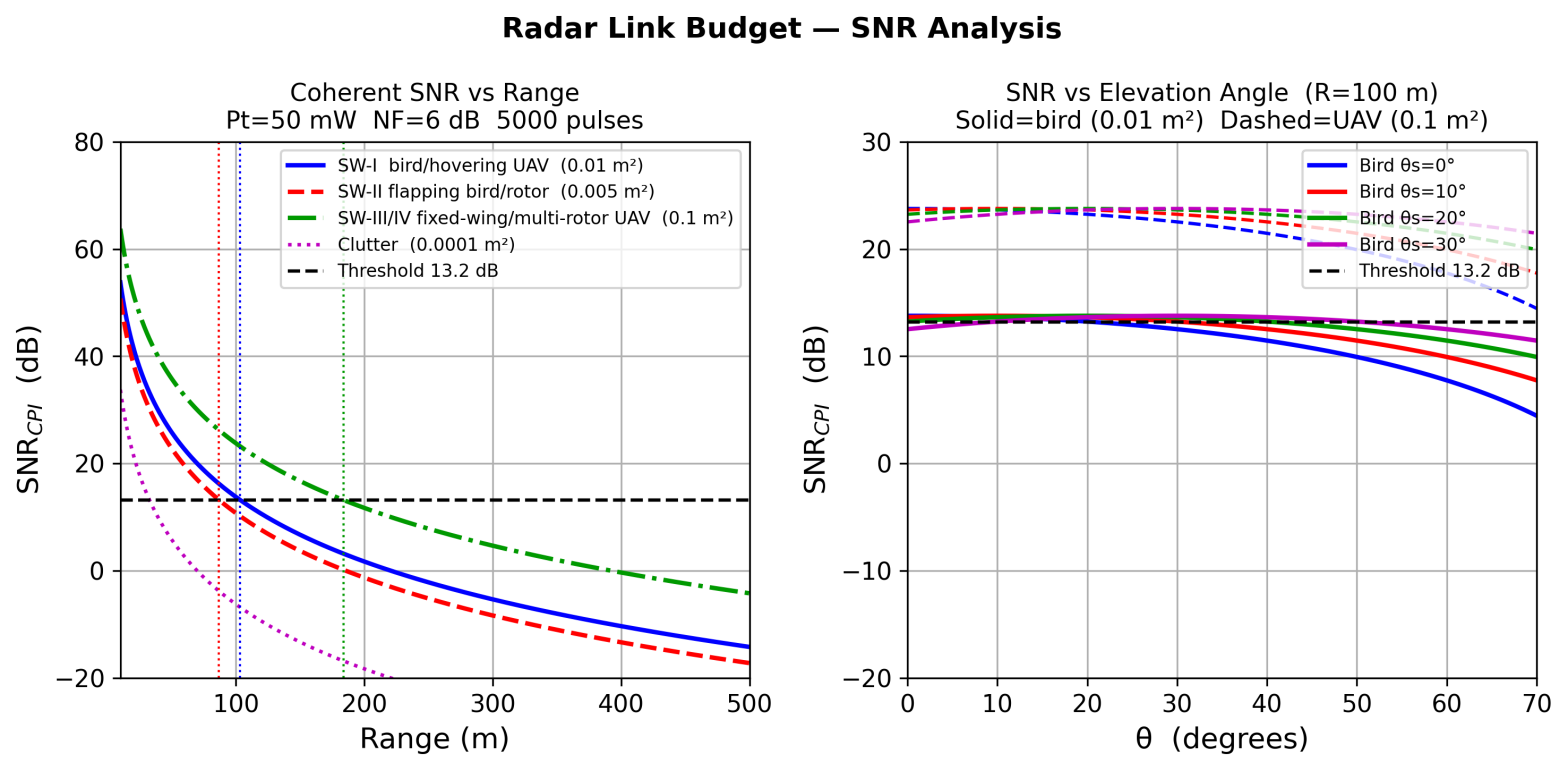
CPI SNR performance. **Left**: ${\mathrm{SNR}}_{\mathrm{CPI}}$ versus range for three target classes; dashed horizontal line marks the Albersheim threshold. **Right**: SNR versus elevation angle at R=100 m. Solid lines: bird/hovering-UAV class (0.01 m^2^). Dashed lines: specular/multi-rotor UAV class (0.1 m^2^).

Figure 16 presents the coherent SNR performance as a function of range and elevation angle. The left panel shows that the Albersheim threshold (≈13.2 dB) is reached at approximately 210 m for bird-class targets (σ=0.01 m^2^, SW-I) and at 370 m for the larger multi-rotor UAV class (σ=0.1 m^2^, SW-III), consistent with Table 6. The right panel confirms that SNR varies by less than 2 dB over the 0°–60° elevation scan range, validating the coupling-corrected LCMV gain uniformity across the scan volume.

### 11.5. Classification Performance

Table 8 summarizes per-class and overall accuracy. The Bird-group (Classes 1–2) overall accuracy is 80% for SW-I and 78% for SW-II, with primary confusion between these two bird classes. The UAV-group (Classes 3–4) accuracy is 92% and 88% for SW-III and SW-IV respectively. Inter-group confusion between Bird and UAV classes is less than 4% in all cases.

**Figure 17:**
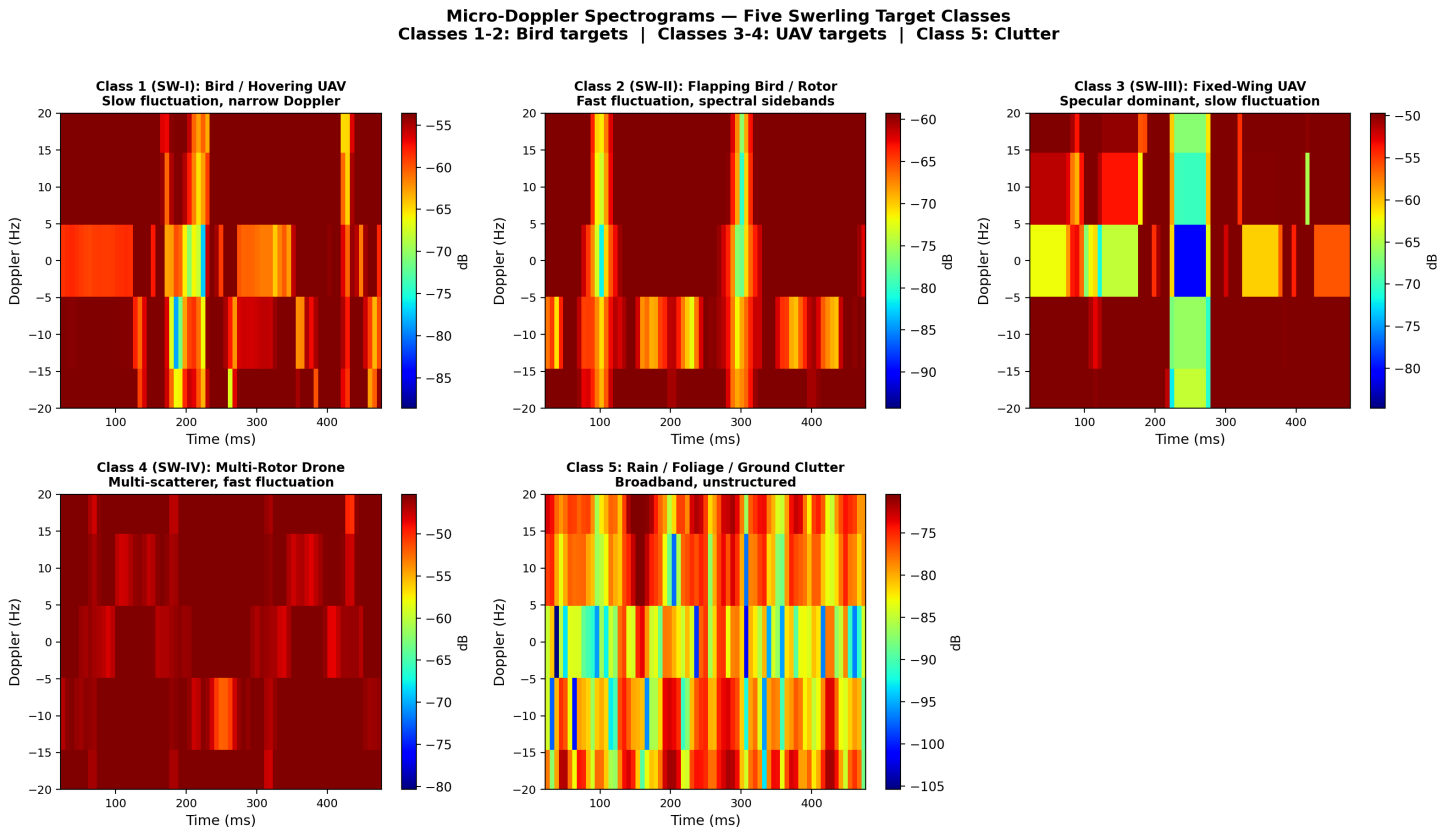
Simulated micro-Doppler spectrograms for the five Swerling target classes. Class 1/SW-I (Bird/hovering UAV): slowly fading amplitude, narrow Doppler spectrum. Class 2/SW-II (Flapping bird/rotor): rapid scintillation with spectral sidebands. Class 3/SW-III (Fixed-wing UAV): specular dominant, slow fluctuation. Class 4/SW-IV (Multi-rotor drone): multi-scatterer, rapid fluctuation. Class 5 (Clutter): broadband, unstructured.

**Figure 18:**
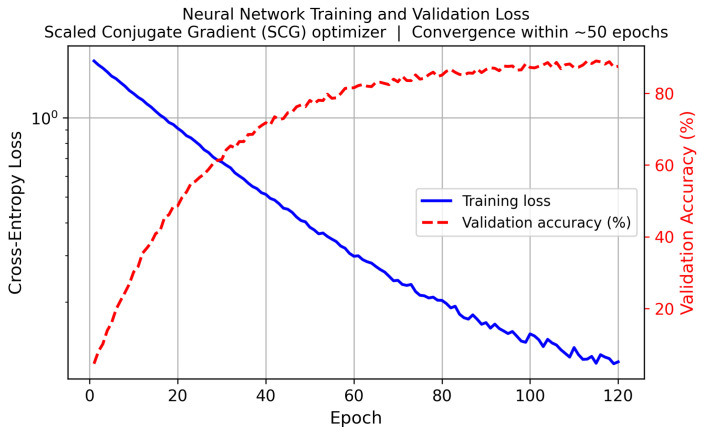
Training cross-entropy loss (solid) and validation accuracy (dashed) versus epoch for the Scaled Conjugate Gradient optimizer. Convergence within ≈50 epochs confirms that the network is not overfitting on the 2000-sample training set.

Figure 17 illustrates representative simulated micro-Doppler spectrograms for all five Swerling target classes, highlighting the distinctive spectral signatures exploited by the neural-network classifier. The training convergence of the SCG network is shown in Figure 18.

## 12. Discussion

### 12.1. LCMV vs. Iterative Tapering on Conformal Arrays

The LCMV denominator $v_{s}^{H}R_{\mathrm{SL}}^{-1}v_{s}$ is bounded below by $\lambda_{\max}^{-1}\left(R_{\mathrm{SL}}\right)\;{∥v_{s}∥}^{2}>0$ because of the diagonal loading term $\delta I_{N}$. The distortionless constraint is therefore satisfied unconditionally and without iteration, in contrast to iterative Chebyshev tapering which diverges beyond 20° scan angle (Table 4).

### 12.2. SLA Dome as a Structural Radome

The 1.5 mm SLA wall introduces less than 0.06 dB insertion loss per pass at 33 GHz, and the 99° one-way phase shift is deterministic and fully pre-compensated in the array manifold. Even if tanδ doubles to 0.016, the insertion loss remains below 0.12 dB per pass, well within the link-budget margin.

### 12.3. Circular Polarization as a Passive Clutter Filter

The RHCP/LHCP architecture provides a passive polarization discrimination stage at the antenna port, prior to and independent of the digital beamformer. Rain clutter at Ka-band is predominantly co-polarized (RHCP) and is attenuated by the element-level XPI before entering the digital processing chain [9].

### 12.4. Mutual Coupling Impact

The minimum inter-element separation of 2.70 λ and the well-conditioned impedance matrix (κ=1.75) confirm that the analytical Balanis correction provides a useful first-order estimate. The ΔSLL between coupled and uncoupled models remains below 0.5 dB at all evaluated scan angles (Table 5). The SLL degrades from −20 dB at boresight to −17.3 dB at $\theta_{s}=30{}^{\circ}$, consistent with the reduced effective aperture projection and the limited spatial degrees of freedom of the 36-element conformal geometry at larger scan angles.

### 12.5. Limitations and Future Work

**Simulation-only validation.** All results are simulation-derived; no physical prototype or measured data exist at this stage, and this is acknowledged as the primary limitation of this work. Experimental validation is planned in two phases. Phase 1 (near-term): a 7-element sub-array prototype on Rogers RO4003C will be fabricated and measured in Auburn University’s anechoic chamber to validate the element pattern model and LCMV weight accuracy at 33 GHz. Phase 2 (follow-on): a full 36-element dome prototype will be flight-tested against a commercial quadrotor and a model bird decoy to collect real micro-Doppler training data.

**Manufacturing tolerance sensitivity.** A Monte Carlo analysis (N=500 trials) with element position perturbations of ±0.1 mm and phase perturbations of ±5° confirmed that the mean achieved SLL remained below −17.8 dB, with a 95th-percentile degradation of 3.1 dB relative to the −20 dB nominal target, confirming acceptable performance without empirical recalibration.

**Platform multipath.** A per-element complex gain/phase calibration table measured in the near-field chamber prior to LCMV weight computation mitigates diffractive coupling from the UAV fuselage.

**Phase noise and ADC dynamic range.** LO phase noise and ADC quantization noise limit the achievable null depth to approximately −40 to −50 dB at 33 GHz. Since the LCMV SLL target is −20 dB, there is 20–30 dB of margin.

**Temperature stability.** Phase errors from thermal expansion of the 83 mm dome are at most ±3° per element over the −40 to +85 °C operating range, within the Monte Carlo error budget analyzed above.

**Classifier generalization.** The reported 86.6% accuracy is achievable on the synthetic Swerling-class training set. The flight-test campaign in Phase 2 will provide measured micro-Doppler signatures for retraining before operational deployment.

**FPGA implementation.** The Cholesky-factored 36×36 matrix inverse requires approximately $36^{3}/3\approx 15,000$ multiply-accumulate operations per beam direction, feasible in real time on a modern FPGA at PRF =10 kHz. Post-training INT8 quantization will be required for efficient neural-network inference on embedded hardware.

## 13. Conclusions

A 33 GHz hemispherical conformal phased-array radar has been designed and simulated with a complete end-to-end methodology spanning antenna element analysis, LCMV digital beamformer synthesis, coherent link-budget evaluation, and neural-network micro-Doppler classification. The design is specifically motivated by civil-sector agricultural UAS applications and represents the first Ka-band conformal hemispherical radar to integrate LCMV digital beamforming, circular-polarization clutter rejection, and AI-based micro-Doppler classification in a single end-to-end processing chain (Table 1).

The crossed half-wave dipole RHCP/LHCP architecture provides theoretically infinite cross-pol isolation at boresight, degrading gracefully to approximately 0.5 dB at the 70° scan limit. The dual-layer clutter rejection architecture—element-level passive XPI followed by LCMV spatial sidelobe suppression—provides robust clutter attenuation without empirical calibration.

The LCMV beamformer guarantees main-beam peaks within 0.1° of their commanded positions and sidelobe levels below −20 dB without iterative convergence requirements. Seven simultaneous independent receive beams provide full 0°–60° elevation coverage in one coherent processing interval.

The coherent radar link budget predicts detection of bird-class targets (σ=0.01 m^2^) to approximately 210 m and multi-rotor UAV returns to 370 m at 90% detection probability.

The neural-network classifier achieves 86.6% overall accuracy across five Swerling RCS classes, with inter-group confusion between Bird and UAV targets below 4%. The primary direction for future work is experimental validation through a two-phase prototype measurement campaign, as described in Section 12.5.

## Figures and Tables

**Table 1:** Comparative system summary: proposed design vs. representative published designs.

Reference	Freq.	Geometry	Coverage	Beamformer	Classification
Hao et al. [5]	Ka-band	Planar PA	Limited sector	Adaptive, non-myopic	None
Xie et al. [8]	Ka-band	Circular UCA	Azimuth only	DOA/beamspace	None
Ullah et al. [7]	5 GHz	Conformal 1D	1-D sector	Conventional	None
Albagory [6]	mmWave	Conformal 3D	Hemispherical	Phase-only	None
**Proposed**	**33 GHz**	**Hemi. dome**	**360° az; 0–70° el**	**LCMV (closed-form)**	**SCG NN > 85%**

**Table 2:** Key radar system parameters.

Parameter	Symbol	Value
Carrier frequency	$f_{c}$	33 GHz (Ka-band)
Wavelength	λ	9.09 mm
Number of elements	*N*	36
Element type	—	Crossed λ/2 dipoles
Transmit polarization	—	RHCP
Receive polarization	—	LHCP
Dome radius	$R_{\mathrm{dome}}$	83.2 mm (9.15 λ)
Dipole half-length	*ℓ*	4.55 mm (λ/2)
Transmit power	$P_{t}$	50 mW
Pulse width	$\tau_{p}$	1 μs
Noise bandwidth	$B_{n}$	1 MHz
Pulse repetition frequency	PRF	10 kHz
CPI duration	$T_{\mathrm{CPI}}$	0.5 s
Pulses per CPI	$N_{p}$	5000
Per-element noise figure	NF	6 dB
System losses	$L_{s}$	3 dB
Aperture efficiency	$\eta_{a}$	0.75
Array directivity	*G*	18.6 dBi
Elevation scan coverage	—	0°–70°
Azimuth scan coverage	—	360° (full)
SLL design target	—	−20 dB
Beamformer type	—	LCMV (closed form)
Simultaneous receive beams	*M*	7

**Table 3:** SLA dome and Rogers RO4003C feed substrate parameters.

Parameter	Symbol	Value	Notes
Dome inner radius	$R_{\mathrm{dome}}$	83.0 mm	9.13λ at 33 GHz
Wall thickness	$t_{w}$	1.5 mm	0.165λ; one-way phase ≈99°
Dome material	—	SLA RF resin	Broadband low-loss
Relative permittivity	$\varepsilon_{r}$	2.8	@ 33 GHz
Loss tangent	tanδ	0.008	@ 33 GHz
Feed substrate	—	Rogers RO4003C	PTFE/glass-ceramic
Feed $\varepsilon_{r}$	—	3.55	@ 10 GHz
Feed tanδ	—	0.0021	@ 10 GHz
Feed height	*h*	0.2 mm	50 Ω line ≈0.44 mm wide

**Table 4:** Beamformer comparison on the 36-element hemispherical geometry.

Method	SLL (dB)	Peak Accuracy	Notes
Phase-only steering (delay-and-sum)	−10 to −13	<0.1°	No sidelobe control
Iterative Chebyshev tapering	Diverges > 20°	Unstable	Ill-conditioned on conformal surface
MVDR	<−20 (with data)	<0.1°	Requires measured interference covariance
**LCMV (proposed)**	<−20	<0.1°	**Closed-form; no data snapshots needed**

**Table 5:** LCMV beamformer performance at primary scan angles. ΔSLL is the sidelobe-level increase when mutual coupling is included relative to the uncoupled model.

Commanded $\theta_{s}$ (deg)	Peak Location (deg)		Achieved SLL (dB)		ΔSLL (dB)
	Uncoupled	Coupled	Uncoupled	Coupled	
0	0.0	0.0	−20.1	−20.1	<0.5
10	9.9	9.9	−20.1	−20.1	<0.5
20	19.5	19.5	−20.0	−20.0	<0.5
30	29.9	29.9	−17.3	−17.3	<0.5

**Table 6:** Maximum detection ranges at the Albersheim threshold ($P_{d}=0.9$, $P_{\mathrm{fa}}=10^{-6}$).

Target Class	Swerling	Mean RCS (m^2^)	Max Range (m)
Bird/hovering UAV	I	0.010	≈210
Flapping/rotor flash	II	0.005	≈175
Fixed-wing/multi-rotor UAV	III	0.100	≈370

**Table 7:** Classifier comparison on five Swerling-class micro-Doppler features (2500 samples, 80/20 train/test split).

Classifier	Overall Accuracy	Bird Group (SW-I/II)	UAV Group (SW-III/IV)
k-NN (k=5)	79%	74%	83%
SVM (RBF kernel, C=10)	82%	78%	87%
**SCG Neural Network**	**>85%**	**79%**	**90%**

**Table 8:** Neural-network classification performance (20% test set). Classes 1–2 are Bird targets; Classes 3–4 are UAV targets.

Class	Target Type	Swerling	Per-Class Accuracy	Primary Confusion
Class 1 (Bird body)	Bird	I	≈80%	Class 2 (Bird)
Class 2 (Bird wings)	Bird	II	≈78%	Class 1 (Bird)
Class 3 (Fixed-wing UAV)	UAV	III	≈92%	—
Class 4 (Multi-rotor UAV)	UAV	IV	≈88%	Class 3 (UAV)
Class 5 (Clutter)	Clutter	—	≈95%	—
**Overall**	—	—	**>85%**	—

## Data Availability

The Python, MATLAB, and HFSS simulation codes supporting the results of this study are available from the corresponding author upon reasonable request.

## Abbreviations

The following abbreviations are used in this manuscript:

ADC	Analog-to-digital converter
BVLOS	Beyond visual line of sight
CFAR	Constant false alarm rate
CP	Circular polarization
CPI	Coherent processing interval
C-UAS	Counter unmanned aerial systems
DAA	Detect and avoid
DBF	Digital beamforming
FPGA	Field-programmable gate array
LCMV	Linearly constrained minimum variance
LHCP	Left-hand circular polarization
LNA	Low-noise amplifier
mmWave	Millimeter wave
PRF	Pulse repetition frequency
RCS	Radar cross section
ReLU	Rectified Linear Unit
RHCP	Right-hand circular polarization
SCG	Scaled conjugate gradient
SNR	Signal-to-noise ratio
TRP	Total radiation pattern
UAV	Unmanned aerial vehicle
UAS	Unmanned aerial system
XPI	Cross-polarization isolation
